# Use and outcomes of antihypertensive medication treatment in the US hypertensive population: A gender comparison

**DOI:** 10.34172/hpp.2023.17

**Published:** 2023-07-10

**Authors:** Shakir Ullah, Shahbaz Khan, Shahrzad Bazargan-Hejazi, Ernesto Ramirez, Senait Teklehaimanot, Sara Diab, Maria Bangash, Magda Shaheen

**Affiliations:** ^1^Charles R. Drew University of Medicine and Science and David Geffen School of Medicine, University of California at Los Angeles, CA, USA; ^2^Ayub Medical College and Teaching Hospital, Abbottabad, Pakistan; ^3^Department of Psychiatry, Charles R. Drew University of Medicine and Science and David Geffen School of Medicine, University of California at Los Angeles, CA, USA; ^4^University of California, Irvine, CA, USA; ^5^Southern California University of Health and Sciences, CA, USA

**Keywords:** Antihypertensive agents, Blood pressure control, Comorbidity, Gender differences, Hypertension, Medication adherence, Racial disparities

## Abstract

**Background:**

Although effective antihypertensive medications have existed for decades, only about half of the hypertensive individuals are considered to have controlled blood pressure. Limited research studies have investigated gender disparity in the utilization and effectiveness of antihypertensive medications treatment. To examine the gender difference in antihypertensive medications’ use and the effect of using antihypertensive medication treatment on blood pressure control among the U.S. adult with hypertension.

**Methods:**

Analysis of National Health and Nutrition Examination Survey (NHANES) data from (1999-2012) including individuals≥18 years old with hypertension. Study variables included gender, age, race/ethnicity, obesity, smoking, comorbidities, treatment medication type, and continuity of care. We used multivariate logistic regression in STATA V14. The data is presented as adjusted odds ratios (ORs) and 95% confidence interval (CI).

**Results:**

Of the 15719 participants, 52% were female. 49% of the antihypertensive medication users had their blood pressure under control (95% CI). In the adjusted logistic regression analysis, use of antihypertensive medications was found to be 12% greater in females as compared to males (OR=1.12; CI=1.02-1.22; *P*<0.05). No association between gender and blood pressure control was found. Blood pressure control was less likely achieved among 50 years or younger individuals, Blacks and Hispanics, obese, and those taking calcium channel blocker (CCB).

**Conclusion:**

Hypertensive females are more likely than males to use antihypertensive medications. The effectiveness of treatment to control blood pressure is equal across males and females. Our findings have implications for practitioners to account gender-specific approaches when discussing adherence to hypertension medication treatment with their patients.

## Introduction

 Hypertension is a well-recognized and modifiable risk factor for cardiovascular diseases (CVD), such as myocardial infarction, stroke, and heart failure, and subsequently CVD mortality.^1,2^ According to the National Center for Health Statistics, the overall prevalence of hypertension among American adults from 2011 to 2016 was approximately 30% for both genders, which is relatively unchanged since 1999.^3-5^ Hypertension that remains untreated adversely impacts individuals’ health and wellbeing as well as raises the treatment cost. In the U.S., the annual healthcare spending related to hypertension was found to be $131 billion when averaged over 12 years, from 2003 to 2012.^6^ It is estimated that individuals with hypertension have annual healthcare costs of $2000, which is greater than their counterparts.^6^ Effective medication treatment to lower blood pressure, such as angiotensin-converting enzyme inhibitors (ACEI), angiotensin receptor blockers (ARBs), diuretics, beta-blockers, and calcium channel blockers (CCBs) have existed for several decades. However, only about 50% of Americans living with hypertension during 2011-2016 had their blood pressure under control.^3-5^ Moreover, there remains an issue with adherence to antihypertensive medications despite their efficacy in controlling hypertension and reducing cardiovascular events.^7,8^ Non-adherence is associated with many factors, including inadequate insurance,^9^ age, race/ethnicity, and income.^8^ Past studies have focused on the relationship between gender and the utilization and effectiveness of antihypertensive medications among the U.S. adults, yet gender-specific guidelines for the treatment of hypertension and blood pressure control goals remain unaddressed.^10,11^ The Systolic Blood Pressure Intervention Trial (SPRINT) elucidated the need to establish the more intensive treatment of hypertension cut-off goals for both genders, like a systolic blood pressure of ≤ 130 mm Hg. However, the optimal blood pressure for both genders was not obtained due to a lack of significant data that stemmed from low female enrollment rates (males 64%, females 36%).^12^ Their finding emphasizes the need to clarify the relationship between gender and hypertension to optimize the potential of reducing high blood pressure and subsequent cardiovascular events. This study aimed to examine the gender difference in antihypertensive medications’ use and the effect of using antihypertensive medication treatment on blood pressure control among the U.S. adult with hypertension.

## Materials and Methods

###  Study design, data and sample

 This cross-sectional study used the National Health and Nutrition Examination Survey (NHANES) data from 1999 to 2012.^13^ The NHANES data is a cross-sectional survey based on a nationally representative sample of the U.S. population and is sponsored by the Center for Disease Control and Prevention. The survey combines an in-person home interview followed by a physical examination, including biomarkers. Non-institutionalized US civilians, aged 18 and older, with hypertension, were eligible to participate in the survey. Following the 8th Joint National (JNC 8) Committee’s recommendation, hypertension was defined as a systolic value ≥ 140 mm Hg or a diastolic value ≥ 90 mm Hg.^14^ A participant was classified having hypertension if he/she met one of the following criteria: 1) had a mean systolic reading of ≥ 140 mm Hg or mean diastolic reading of ≥ 90 mm Hg measured during the physical examination. 2) The participant had been on antihypertensive medication within the last 30 days. 3) The participant recalled being previously diagnosed with hypertension by a physician.

###  Study variables

 The physical examination for NHANES data took place at a local mobile examination center and included measurements of the participants’ blood pressure, height and weight, and blood and urine tests. Trained physicians following standard protocol obtained blood pressure measurements after the participants rested for five minutes in a seated, upright position. Mid-arm circumference was used to determine the appropriate cuff size for each participant. Three consecutive blood pressure readings were obtained, 30 seconds apart, and an average was calculated to determine the mean blood pressure for each participant. The outcome variables for the study were taking medications for blood pressure and subsequent controlled blood pressure. Blood pressure medication use and type were obtained from the participants during the home interview when they were asked “have you taken or used any prescription medicines for blood pressure in the past month?” If the participant answered yes to this question, the name, duration, and primary reason for each medication was obtained. Antihypertensive medications reported by the participants were classified into one of five categories: diuretics, ACE inhibitors, ARBs, beta-blockers, CCBs, and other antihypertensive medications (including α1-blockers, α2-agonists, and vasodilators). Blood pressure control was defined as a blood pressure reading of less than 140/90 mm Hg and was measured during the physical examination at the local mobile examination center. The primary predictor variable was gender, categorized as male or female. Other independent variables in the study include age (18-39; 40-49; 50-59; 60-69; 70-79, and ≥ 80 years); race/ethnicity (White, Black, and Hispanic); comorbidities including diabetes, chronic kidney disease, congestive heart failure (CHF), stroke, and coronary heart disease, obesity based on body mass index (BMI) (obese vs. non-obese); smoking status (former smoker, current smoker, or non-smoker); continuity of care, and antihypertensive medication type.

###  Data analysis

 A descriptive statistical analysis was performed to determine the general characteristics of the study population. Categorical variables, such as age groups, were described with numbers and percentages, while continuous variables, such as blood pressure, were described with means and standard deviations. A bivariate statistical analysis was subsequently performed to display the study demographics by gender (male vs. female) using chi-square tests. A two-step multivariate statistical analysis was performed using adjusted logistic regressions. First, we identified predictors of antihypertensive medication use in the hypertensive participants. Then, we analyzed the status of blood pressure control among medication users. In both analyses, we controlled for the demographics and other characteristics of the study sample. We used adjusted odds ratios (ORs) and 95% confidence interval (CI) to report our findings and STATA V14®^15^ statistical software to analyze the data.

## Results

Table 1 summarizes the demographic results of 15 719 individuals from 1999 to 2012. Both genders were represented almost equally: 48% males and 52% females. Individuals < 60 years old accounted for 56% of the study population. Of the participants, Blacks and Hispanics made up 14% and 9%, respectively. Diabetes was the most common comorbidity (16%), followed by congenital heart disease (CHD) (7%), stroke (6%), CHF (5%), and kidney disease (4%). Both ACE inhibitors and beta-blocker were found to be the most commonly prescribed antihypertensive medications (21% individually), followed by diuretics (17%), CCB (16%), and ARB (9%). The mean systolic blood pressure reported was 135 mm Hg ± 20.3, while the mean diastolic blood pressure was 74 mm Hg ± 15.4.

**Table 1:** Sample demographics by males, and females (N = 15 719)

**Variable**	**Total** **(N=15719)**	**Male** **(N=7545)**	**Female** **(N=8174)**	***P*** ** value**
	No. (%)*	No. (%)*	No. (%)*	
Age				0.01
18-39	2515 (15.9)	1433 (18.9)	980 (11.9)	
40-49	2829 (17.9)	1433 (18.9)	1308 (15.9)	
50-59	3458 (21.9)	1659 (21.9)	1798 (21.9)	
60-69	3143 (19.9)	1433 (18.9)	1553 (18.9)	
70-79	2357 (14.9)	980 (12.9)	1063 (12.9)	
≥ 80	1414 (8.9)	452 (5.9)	490 (5.9)	
Gender				
Male	7545 (47.9)	-	-	-
Female	8174 (52.0)	-	-	
Race/ethnicity				
White	11317 (71.9)	5507 (72.9)	5805 (70.9)	0.01
Black	2200 (13.9)	905 (11.9)	736 (8.9)	
Hispanic	1414 (8.9)	679 (8.9)	1226 (14.9)	
Other	785 (4.9)	377 (4.9)	408 (4.9)	
Smoking				
Current	2986 (18.9)	1659 (21.9)	1389 (16.9)	0.01
Former	5030 (31.9)	2867 (37.9)	2125 (25.9)	
Non-smoker	7702 (48.9)	3018 (39.9)	4659 (56.9)	
Obesity				
Obese	7230 (45.9)	3244 ( (42.9)	3923 (47.9)	0.01
Non-obese	8488 (53.9)	4300 (56.9)	4250 (51.9)	
Continuity of care				
Yes	14461 (91.9)	6715 (88.9)	7765 (94.9)	0.01
Co-Morbidity				
Diabetes	2515 (15.9)	1131 (15.17)	1389 (16.9)	0.10
Congestive heart failure	785 (4.9)	377 (4.9)	408 (4.9)	0.74
Chronic heart disease	1100 (6.9)	679 (8.9)	408 (4.9)	0.01
Stroke	943.14 (6)	377 (4.9)	572 (6.9)	0.01
Kidney disease	628 (3.9)	226 (2.9)	408 (4.9)	0.01
Treatment type				
ACEI	3300 (20.9)	1584 (20.9)	1634 (19.9)	0.11
Beta blockers	3300 (20.9)	1584 (20.9)	1716 (20.9)	0.28
Calcium channel blockers	2515 (15.9)	1056 (13.9)	1389 (16.9)	0.03
Diuretics	2672 (16.9)	1131 (14.9)	1553 (18.9)	0.01
ARB	1414 (8.9)	603 (7.9)	735 (8.9)	0.01
Blood pressure (Mean ± SD)	135 ± 20.3	136 ± 20	138 ± 23	0.01
Systolic				
Diastolic	74 ± 15.4	74 ± 16	70 ± 16	

*Numbers do not add up due to missing data. ACEI, Angiotensin converting enzymes inhibitors; ARB, Angiotensin receptors blockers.

Table 1 also displays the association between study characteristics by gender. Males had a higher percentage of hypertension in the 18 to 48 age groups, but a lower percentage in the ≥ 60 groups, relative to females. Females, in comparison to males, had a higher percentage of obesity, stroke and kidney disease, and continuity of care (*P* < 0.01). Moreover, higher proportion of females in compared to males used CCBs (17% vs. 14%), diuretics (19% vs. 15%), and ARBs (9% vs. 8%) (*P* < 0.01). Females had a higher mean systolic blood pressure, while males had a higher mean diastolic blood pressure (*P* < 0.01).

Table 2 displays univariable logistic regression of the independent predictors of hypertensive medication use among the hypertensive population (N = 14 795). Females, compared to males, were more likely to be on hypertensive medications (OR = 1.12, 95% CI = 1.02-1.22, *P* < 0.01). Other independent predictors of using hypertensive medications were being 40 and older in comparison to the younger age groups (*P* < 0.01); being former smoker (OR = 1.26, 95% CI = 1.09-1.46) or non-smoker (OR = 1.21, 95% CI = 1.07-1.38) in compared to the current smokers, and being obese compared to non-obese (OR = 1.25, 95% CI = 1.14-1.38). No statistical significance was found between race and being hypertensive in the sample population.

**Table 2:** Independent predictors of using hypertensive medication among hypertensive population (n = 14 795)

**Hypertensive population (N=14795)**		
**Variable**	**OR (95% CI)**	***P*** ** value**
Age		
18-39	Reference	
40-49	1.77 (1.46-2.15)	0.01
50-59	2.28 (1.95-2.67)	0.01
60-69	2.76(2.31-3.29)	0.01
70-79	3.11(2.58-3.74)	0.01
≥ 80	3.40(2.67-4.32)	0.01
Gender		
Male	Reference	
Female	1.12(1.02-1.22)	0.01
Race/ethnicity		
White	Reference	
Black	1.03(0.93-1.14)	0.55
Hispanic	0.86(0.73-1.02)	0.09
Others	1.16(0.93-1.44)	0.19
Smoking		
Current	Reference	
Former	1.26(1.09-1.46)	0.01
Non-smoker	1.21(1.07-1.38)	0.01
Obesity		
Non-obese	Reference	
Obese	1.25(1.14-1.38)	0.01
Continuity of care		
No	Reference	
Yes	1.13(0.97-1.33)	0.12
Co-morbidity		
No diabetes	Reference	
Diabetes	1.69(1.47-1.93)	0.01
Co-Morbidity		
No CHF	Reference	
CHF	1.57(1.22-2.01)	0.01
Co-Morbidity		
No CHD	Reference	
CHD	2.00(1.58-2.52)	0.01
Co-morbidity		
No stroke	Reference	
Stroke	1.45(1.18-1.78)	0.01
Co-morbidity		
No kidney disease	Reference	
Kidney disease	1.33(1.02-1.73)	0.04

OR, odds ratio; CI, confidence interval; CHF, congestive heart failure; CHD, chronic heart disease.

 Also, participants with comorbidity, including diabetes, CHF, CHD, stroke (*P* < 0.01), and kidney disease (*P* < 0.04), had higher odds of being on hypertensive medications compared to their counterparts without any comorbidity. These findings show a strong relation between hypertension and co-morbidities at confidence interval of 95%.

Table 3 illustrates the independent predictors of having controlled blood pressure (blood pressure less than 140/90 mm Hg) among medication users. Of the antihypertensive medication users (N = 14 795), 7753 individuals (49%) had their blood pressure under control. We did not find any statistically significant difference between hypertensive medication use and controlled blood pressure when comparing females to males. Compared to the 18-39 age group, 50-59, 60-69, 70-79, and ≥ 80 age groups had lower odds of having controlled blood pressure (*P* < 0.05). Furthermore, compared to the Whites, Blacks and Hispanics had lower odds of having their blood pressure controlled (32% and 18%, respectively) (*P* < 0.01). Obese individuals were 1.23 times at greater odds of having controlled blood pressure than non-obese participants (*P* < 0.01). Also, individuals taking CCB were 14% less likely to have their blood pressure controlled than individuals not taking CCB (*P* < 0.05).

**Table 3:** Independent Predictors of Having Controlled Blood Pressure among Medication Users (N = 7753)

**Hypertensive Population (N=7753)**		
**Variable**	**OR (95% CI)**	***P*** ** value**
Age		
18-39	Reference	
40-49	0.92(0.70-1.21)	0.55
50-59	0.75(0.57-0.99)	0.04
60-69	0.57(0.44-0.75)	0.01
70-79	0.47(0.36-0.61)	0.01
≥ 80	0.29(0.22-0.39)	0.01
Gender		
Male	Reference	
Female	0.96(0.84-1.10)	0.56
Race/ethnicity		
White	Reference	
Black	0.68(0.61-0.78)	0.01
Hispanic	0.82(0.70-0.96)	0.01
Other	0.83(0.63-1.09)	0.18
Smoking		
Current	Reference	
Former	0.99(0.82-1.20)	0.93
Non-smoker	0.85(0.69-1.02)	0.08
Obesity		
Non-obese	Reference	
Obese	1.23(1.09-1.39)	0.01
Continuity of care		0.36
No	Reference	
Yes	1.12(0.88-1.41)	--
Co-Morbidity		0.56
No diabetes	Reference	
Diabetes	1.04(0.90-1.21)	-
Co-Morbidity		0.16
No congestive heart failure	Reference	
Congestive heart failure	1.10(0.88-1.38)	--
Co-Morbidity		0.40
No chronic heart disease	Reference	
Chronic heart disease	1.10(0.88-1.38)	--
Co-Morbidity		0.21
No Stroke	Reference	
Stroke	0.86(0.68-1.09)	--
Co-Morbidity		0.75
No kidney disease	Reference	
Kidney disease	0.96(0.75-1.23)	--
Treatment		
No ACEI	Reference	
ACEI	1.05(0.90-1.23)	0.52
Treatment		
No beta-blocker	Reference	
Beta blocker	0.93(0.79-1.08)	0.34
No calcium channel blockers	0.86(0.74-0.99)	0.34
Calcium channel blockers	0.86(0.74-0.99)	0.34
Treatment		
No diuretics	Reference	
Diuretics	1.11(0.96-1.28)	0.15
Treatment		
No ARB	Reference	
ARB	1.06(0.88-1.27)	0.57

CI, confidence interval; ACEI, angiotensin-converting enzyme inhibitors; ARB, angiotensin II receptor blockers.

## Discussion

 Our study found that females were more likely to use antihypertensive medications relative to males. Moreover, we did not find any significant differences in blood pressure control between males and females. Our finding of gender differences parallels a previous study that analyzed the NHANES data set between 1999 to 2004 found women used antihypertensive mediations at a higher prevalence 61.4%, relative to men 56.8%.^16^ These results may be explained by consistent gender differences noted in the literature regarding the utilization of health services and the use of medications. A continuous trend in the literature shows that women use health services more than men. Specifically, one study noted that women had a significantly higher number of mean visits to their primary care clinic and diagnostic services ordered along with higher associated medical service charges.^17^ Also, studies have found that women use more medications, across various medication types and disease states, than men.^18,19^ Thus, the higher odds of women using antihypertensive medications extend the current literature with an emphasis on hypertension. This finding may suggest the need for gender-specific approaches when discussing hypertension with patients to mitigate the gender difference seen in medication use. Thus, further studies should focus on the factors associated with or causing the reported gender disparity.

 Additionally, we found that former smokers and lifetime non-smokers were more likely to be on antihypertensive medications than current smokers. Current smokers may avoid seeking medical attention to avoid talking to physicians regarding quitting smoking. Moreover, hypertensive individuals who were obese or had comorbidities had a higher likelihood of being on medications for hypertension. These results may allude to the possibility that these individuals were sicker with other conditions and were already seeing a physician for those conditions and were already taking antihypertensive mediations medications related to their respective comorbidities. For example, individuals with CHF and Stroke were probably on antihypertensive medications due to having a history or CHF and stroke.

 Lack of any significant differences in blood pressure control between males and females parallels with some of the current literature. One study noted that almost all large, prospective, and randomized trials in the hypertension arena had found no significant gender differences in blood pressure reductions.^20^ A study that used NHANES data from 1999 to 2004 and adjusted logistic regression, similar to the current study, found that blood pressure control rates were not significantly different between genders.^21^ Conversely, a study that also used NHANES data from 1999 to 2004 and adjusted logistic regression found hypertensive women to be less likely to achieve blood pressure control- 44.8% of women were noted to reach blood pressure control compared to 51.1% of men.^16^ One contributing factor to the discrepancy between these two studies may be the larger sample size of the latter study (N = 5410) compared to the former (N = 3475), which may have unveiled the difference between genders within the same timeframe. Other prior studies have varying results compared to our study as they have noted gender differences in blood pressure control- either women having higher rates of control^22,23^ or lower rates of control compared to men.^24,25^ Our findings that there are no gender differences in blood pressure control varies by study design, time-period, sample size, and data analysis, which provides a potential explanation to the conflicting results. These overall inconsistent findings throughout the literature emphasize the need for future studies to evaluate whether gender difference exists in blood pressure control.^26^ By doing so, healthcare practitioners can establish gender directed treatment plans for their hypertensive patients.

 Additionally, Blacks and Hispanics were less likely, 68% and 82% respectively, to have controlled blood pressure when taking medications compared to Caucasians. This is consistent with current research findings^27,28^ and calls for the identification of multifactorial causes.^29^ Furthermore, there was no statistically significant difference between the use of different antihypertensive medications and blood pressure control, expect for CCB. The antihypertensive medication users who were taking CCB were less likely to achieve the targeted blood pressure than individuals not taking CCB.

 Although this study relies on national data and has a large sample size, some limitations should be noted. While the NHANES methods to obtain blood pressure measurements followed national standard protocols and obtained three readings, each participants’ blood pressure was obtained in one sitting time rather than two or more occasions. This may have resulted in inaccurate participant blood pressure classifications. Moreover, to minimize recall bias, NHANES asked participants to recall antihypertensive medications taken in the past month. Therefore, participants who took antihypertensive medications prior to that one month window were classified as non-medication users and potentially non-hypertensive if they did not meet the other two criteria. Thus, the prevalence of hypertension among U.S. adults may have been underreported

## Conclusion

 Our study concluded that, compared to males, females were more likely to adhere to the antihypertensive medication regimen. However, there were no gender differences in reaching the targeted blood pressure (i.e., < 140/90 mm Hg) among hypertensive individuals who were taking antihypertensive medication. Additionally, individuals older than 40 years, smokers, obese, and individuals with co-morbidity were more likely to use antihypertensive medication. However, blood pressure control was less likely to be achieved among hypertensive individuals who were 50 years or younger, Blacks and Hispanics, obese, and those taking CCB. Our findings suggest that providers may consider gender-specific approaches when discussing adherence to antihypertensive medication with their patients to mitigate gender disparity. Further studies are needed to verify our findings and perhaps shed light on why race-ethnic disparities in controlling hypertension continue to exist in the US.

## Acknowledgments

 The research team would like to acknowledge the administrative and editorial support provided by Kaveh Dehghan, MBA.

## Competing Interests

 Shahrzad Bazargan-Hejazi is an associate editor of *Health Promotion Perspective*. Other authors declare no conflicts of interest in this work.

## Data Availability Statement

 Data for this study is publically available for the readers.

## Ethical Approval

 Not Applicable.

## Funding

 Research for this article was supported in part by NIH Accelerated Excellence in Translational Sciences (AXIS) grant number 2U54MD007598-07; and the University of California at Los Angeles (UCLA) Clinical and Translational Science Institute (CTSI), grant number UL1TR001881.
